# Effect of Ultrasonic Treatment on the Quality of Pumpkin Juice Fermented by Yeast

**DOI:** 10.3390/foods14132284

**Published:** 2025-06-27

**Authors:** Wenhui Pan, Wen Li, Chunli Zhou, Manjun Zhang, Wei Su, Renqin Tan, Leyi Yao

**Affiliations:** School of Life Science, Jiangxi Science & Technology Normal University, Nanchang 330013, China; 18120869696@163.com (W.P.); lw18213347282@163.com (W.L.); 13803451806@163.com (M.Z.); suwei74@hotmail.com (W.S.); tanrenqin0807@163.com (R.T.); 15347527171@163.com (L.Y.)

**Keywords:** yeast, fermentation, ultrasonic wave, pumpkin juice, quality

## Abstract

To investigate the effect of ultrasound on the quality of pumpkin juice fermented by yeast, ultrasound (power range 0–400 W, duration 10 min) was used to assist the yeast fermentation of pumpkin juice. The effects of ultrasound-assisted fermentation at different powers on the quality characteristics of pumpkin juice, such as the color, aroma components, carotenoid content, and antioxidant activity, were studied. The optimal combination of fermentation process parameters was determined as follows: a pumpkin juice content of 20 mL, fermentation temperature of 30 °C, fermentation time of 1 day, and inoculation amount of 3% (the mass-to-volume ratio of yeast to water). The results showed that after ultrasound treatment, the physicochemical properties of pumpkin juice, including the Vitamin C (VC) content, carotenoid content, and antioxidant activity, did not change significantly (*p* > 0.05), while the total acid content decreased significantly, indicating that ultrasound treatment was beneficial for improving the sensory properties of pumpkin juice after yeast fermentation. Notably, when the ultrasound power was 100 W, the flavonoid content in pumpkin juice reached the highest level (1.27 mg/100 g). A total of 127 volatile substances were identified via gas chromatography, among which 14 were characteristic aroma profiles of pumpkin juice, suggesting that ultrasound (US) treatment had little impact on the natural flavor of pumpkin juice. Cavitation caused cell rupture in pumpkin juice, and ultrasound treatment significantly improved the sterilization rate of yeast-fermented pumpkin juice and was extremely effective in maintaining its color. This study provides a theoretical basis for the development of high-quality and nutrient-rich fermented pumpkin juice.

## 1. Introduction

Pumpkin, an important vegetable crop, is rich in nutrients and possesses significant healthcare and medicinal values. As a functional food, it is widely applied in industries such as the baking, beverage, meat, and dairy industries. Yeast, a single-celled fungus with unique biological fermentation capabilities, is commonly used as a natural leavening agent in the food industry. Yeast fermentation technology can not only significantly improve the structural properties of food, but also enhance the aroma compounds, leading to remarkable improvements in both food quality and flavor. For instance, Rahman’s study has shown that probiotic-fermented fruit and vegetable juices exhibit higher antioxidant activity and an overall increase in phenolic compound content. Guanwen Suo [1] et al. found that the physical and chemical properties of ultrasonic-treated pumpkin juice were better preserved during storage compared to untreated samples. Therefore, ultrasonic treatment demonstrates broad application prospects in preserving bioactive substances and physicochemical properties, as well as extending the shelf life of fresh pumpkin juice. Consequently, investigating the ultrasound-assisted yeast fermentation of pumpkin juice holds significant benefits for multiple industrial sectors.

Ultrasonic-assisted fermentation technology has emerged out of requirement and demonstrated great potential. In the deep processing of fruits and vegetables, ultrasonic waves can exert a variety of positive effects during the yeast fermentation process [2]. The cavitation effect of ultrasonic waves can disrupt the cellular structure of fruits and vegetables, making the nutrients inside the cells more readily soluble and thus easier to leach out [3]. The disruption of the cellular structure of fruits and vegetables via the cavitation effect of ultrasonic waves provides yeast with a more abundant fermentation substrate, thereby promoting the fermentation process. Some researchers, such as Keșa [4], have discovered that ultrasonic waves can significantly enhance the extraction of substances like phenols, anthocyanins, and aromatic compounds. In this study, the ultrasonic-assisted fermentation of pumpkin juice was carried out to reveal the potential advantages of ultrasonic technology and fermentation technology in improving the quality of pumpkin juice products, enhancing the sterilization effect, promoting the fermentation process, and improving the texture of foods. Our deep exploration of the process parameters and action mechanisms of ultrasonic-assisted fermentation technology provides a new development direction for the deep processing industry of fruits and vegetables and promotes the research, development, and promotion of healthy foods.

## 2. Materials and Methods

### 2.1. Materials and Reagents

The ultrasonic cell disruptor (SCIENTZ-IID) and the UV spectrophotometer (UV-5200PC) (Youke Instrument Co., Ltd., Shanghai, China). Oxalic acid solution, plate count agar (PCA), sodium chloride, anhydrous ethanol, n-hexane, sodium phosphate buffer, catechol solution, NaNO_2_ solution, Al(NO_3_)_3_ solution, DPPH-methanol solution, and toluidine blue were all procured from Solarbio Science & Technology Co., Ltd., Beijing, China. divinylbenzene/polydimethylsiloxane, DVB/CAR/PDMS-50/30, (Supelco, Inc., Bellefonte, PA, USA)The juicer was a Joyoung L18-P132 model (made in China), and the inverted optical microscope (IX-71) (Olympus Corporation, Tokyo, Japan).

### 2.2. Experimental Methods

#### 2.2.1. Preparation of Pumpkin Juice

*Cucurbita moschata* was purchased from the local market in Nanchang City, Jiangxi Province. High-quality pumpkin samples with a bright color and good storability were selected, thoroughly washed, peeled, deseeded, and the pulp was weighed as 600 g. The pulp was cut into small pieces, steamed until soft, cooled, mixed with 600 mL of pure water, and juiced using a juicer.

#### 2.2.2. Ultrasonic Treatment of Pumpkin Juice

A 100 mL sample of pumpkin juice was placed in a 250 mL beaker for SCIENTZIID ultrasonic cell disruptor (Youke Instrument Co., Ltd., Shanghai, China) under the conditions of 25 kHz and 100, 200, 300, and 400 W, for 10 min at 25(±2) °C.

#### 2.2.3. Single-Factor Experiment

In this study, the selection of optimization ranges for the response surface methodology was based on the results of preliminary single-factor experiments. First, four main factors with significant effects on the yeast fermentation of pumpkin juice were initially screened through pre-experiments: pumpkin juice content, yeast inoculation amount, fermentation time, and fermentation temperature. To determine the reasonable level ranges for each factor, further single-factor experiments were conducted. Specifically, the pumpkin juice content was set at five levels: 10%, 15%, 20%, 25%, and 30%; the yeast inoculation amount was set at five gradients: 1 mL, 2 mL, 3 mL, 4 mL, and 5 mL; the fermentation time was set as 0 d, 1 d, 2 d, 3 d, and 4 d, respectively; and the fermentation temperature was set at five levels: 26 °C, 28 °C, 30 °C, 32 °C, and 34 °C (samples were placed in an incubator, and the incubator temperature was adjusted). Each treatment was replicated three times, with the pH value and sensory score as evaluation indicators. The analysis was performed using Design-Expert 10.0 software. Through combined experiments on various factors at different levels, the effects of each factor and their interactions on pumpkin juice fermentation were systematically studied, providing a scientific basis for optimizing the fermentation process.

#### 2.2.4. Determination of the Optimal Fermentation Parameters for Yeast Fermentation of Pumpkin Juice

Based on the analysis of the single-factor experiment results, four factor levels were designed. For the content of pumpkin juice, the levels were set at 15 mL, 20 mL, and 25 mL. The inoculation amount of yeast was set at 2%, 3%, and 4%. The fermentation time was set at 0 day, 1 day, and 2 days. As for the fermentation temperature, the levels were 15 °C, 20 °C, and 25 °C. These factor levels are presented in Table 1 and Table 2.

#### 2.2.5. Determination of Total Acid Content of Pumpkin Juice Fermented by Yeast with Ultrasonic Assistance

The total acid content was calculated according to the method described by Rawson [5] et al. According to the possible content of total acid in the sample, the operator used a pipette to suck 25 mL, 50 mL, or 100 mL of the test solution and placed it in a 250 mL Erlenmeyer flask. Then, 2–4 drops of (10 g/L) phenolphthalein indicator solution were added, and the solution was titrated with a 0.1 mol/L sodium hydroxide standard titration solution until a faint pink color appeared and did not fade within 30 s. Finally, the volume value of the 0.1 mol/L sodium hydroxide standard titration solution consumed was recorded. The calculation formula was as follows:

(1) $$x=\frac{[c\times (V1\times V2)]\times K\times F}{m}\times 1000$$

X—The content of total acid in the sample, with the unit of gram per kilogram (g/kg) or gram per liter (g/L);

c—The concentration of the sodium hydroxide standard titration solution, with the unit of mole per liter (mol/L);

V1—The volume of the sodium hydroxide standard titration solution consumed during the titration of the test solution, with the unit of milliliter (mL);

V2—The volume of the sodium hydroxide standard titration solution consumed during the blank test, with the unit of milliliter (mL);

k—The conversion coefficient of the acid; citric acid is 0.064;

F—The dilution factor of the test solution;

m—The mass of the sample, with the unit of gram (g) or the volume of the sample sucked, with the unit of milliliter (mL);

1000—The conversion coefficient.

The calculation result was expressed as the arithmetic mean of the three independent determination results obtained under repeatability conditions, and the result was retained with two decimal places.

#### 2.2.6. Determination of the Color of Pumpkin Juice Fermented by Yeast with Ultrasonic Assistance

The method for determining the color referred to the approach proposed by Bhutkar [6] et al. The color of the pumpkin juice was determined via spectrophotometric colorimetry (X-Rite, Color i7, US). The test conditions were as follows: total transmission, LAV, D65/10°, and SPIN. The values of L* (lightness/darkness), a* (redness/greenness), and b* (yellowness/blueness) were measured. According to the following formulas, the chroma (C*), hue angle (h^0^), total color difference (Δ*E*), browning index (BI), yellowness index (YI), and color index (CI) were calculated:

(2) $$c^{*}=\sqrt{{\left(a^{*}\right)}^{2}+{\left(b^{*}\right)}^{2}}$$

(3) $$h^{0}=\arctan\left(b^{*}/a^{*}\right)$$

(4) $$\Delta E=\sqrt{{\left(\Delta L^{*}\right)}^{2}+{\left(\Delta a*\right)}^{2}+{\left(\Delta b*\right)}^{2}}$$

BI = 100(*x* − 0.31)/0.172, *x* = (a* + 1.75b*)/(5.645L* + a* − 3.012b*) (5)

YI = 142.86 b*/L* (6)

CI = (180 − h^0^)/(L* − C*) (7)

#### 2.2.7. Determination of the Carotenoid Content in Pumpkin Juice Fermented by Yeast with Ultrasonic Assistance

The method for determining the carotenoid content referred to the approach proposed by João Gustavo Provesi et al. [7] First, 0.2 g of pumpkin juice was weighed and 8 mL of ethanol along with 6 mL of n-hexane were added. The test tube was covered with aluminum foil. Then, the test tube was placed in crushed ice and shaken for 1 h, after which 2 mL of distilled water was added, and the mixture was left to stand for 5 min. All samples were allowed to stand for 10 min, after which the samples in the n-hexane phase were measured using a spectrophotometer at 444, 50, 451, and 472 nm. The calculation formula for the content of carotenoids was:

(8) $$\mathrm{ug}/g=(A_{\mathrm{nm}}\;\times \;M.\mathrm{wt}\;\times \;3)/(0.2\;\times \;E\begin{array}{c} 1\%\\ 1\mathrm{cm} \end{array})$$

The absorbance value was represented by *A*_nm_, the molecular weight of the carotenoids was denoted by M.wt, the volume of the hexane layer was measured as 3 mL, the pumpkin juice was weighed at 0.2 g, and the extinction coefficient of carotenoids in hexane was represented by $E\begin{array}{c} 1\%\\ 1\mathrm{cm} \end{array}$. We used the extinction coefficients $E\begin{array}{c} 1\%\\ 1\mathrm{cm} \end{array}$ of β-carotene, α-carotene, β-cryptoxanthin, zeaxanthin, and lycopene in n-hexane, which were 2560, 2800, 2460, 2480, and 3540, respectively. To convert the result from ug/g to mg/L, appropriate unit conversion factors needed to be applied according to the density of the solution and other relevant parameters. After conversion, the concentration (mg/L) could be calculated.

#### 2.2.8. Determination of Ascorbic Acid in Pumpkin Juice Fermented by Yeast with Ultrasonic Assistance

The method for determining the ascorbic acid referred to the approach proposed by Francisco J. Barba et al. [8]. First, 5 mL of the pumpkin juice sample was taken, 10 mL of distilled water was added, and then it was diluted to a 50 mL volumetric flask with a freshly prepared 2% oxalic acid solution. The sample to be tested was decolorized with activated carbon. Finally, 10 mL of the sample was taken and titrated with the standardized sodium 2,6-dichloroindophenolate until a pinkish red color appeared and did not fade within 15 s. The calculation formula was as follows:

(9) $$W=\frac{V\times A}{B}\times \frac{b}{a}\times 100\%$$

V—The volume of sodium 2,6-dichloroindophenolate used for titrating the sample, in mL;

A—The number of milligrams of ascorbic acid equivalent to 1 mL of sodium 2,6-dichloroindophenolate, in mg;

B—The volume of the sample solution taken during titration, in mL;

b—The total volume in milliliters of the diluted sample;

a—The volume of the sample, in mL;

W—The amount of Vc (ascorbic acid) in 100 mL of fruit and vegetable juice, in mg.

#### 2.2.9. Determination of Flavonoids in Pumpkin Juice Fermented by Yeast with Ultrasonic Assistance

The sample was determined using a slightly modified version of the method described by Kwaw et al. [9]. Specifically, 1 mL of the sample was taken, followed by the addition of 1 mL each of 5% NaNO_2_ solution and 10% Al(NO_3_)_3_ solution. Then 10 mL of a 4% NaOH solution was added, the mixture was shaken thoroughly, diluted to 25 mL with absolute ethanol, allowed to stand for 15 min, and the absorbance was measured at 510 nm. The content of total flavonoids in the sample was expressed in terms of the rutin equivalents.

#### 2.2.10. Determination of the Antioxidant Activity of Pumpkin Juice Fermented by Yeast with Ultrasonic Assistance

The antioxidant activity was determined using a method referenced from the approach proposed by Xu et al. [10], and the DPPH assay method was adopted. First, 0.2 mL of the sample to be tested was taken and mixed with 1.8 mL of a 0.004% DPPH–methanol solution. The mixture was kept at room temperature (25 ± 1) °C for 30 min, and the absorbance at 517 nm was measured and recorded as A_A_. A blank test was performed using a 0.004% DPPH–methanol solution, and the result was recorded as AC. Each test was measured three times. The calculation formula for the DPPH radical scavenging rate was as follows:

(10) $$\mathrm{The}\;\mathrm{scavenging}\;\mathrm{rate}\;\mathrm{of}\;\mathrm{DPPH}\;\mathrm{radicals}/\%=\left(1-A_{A}/A_{C}\right)\times 100$$

#### 2.2.11. Determination of the Total Number of Microorganisms and Their Microscopic Structures in Yeast Fermentation Assisted by Ultrasonic Waves

The method for determining the total number of microorganisms was as follows: the pumpkin juice fermented with the assistance of ultrasonic waves was diluted, spread on Rose Bengal medium, and the culture medium plates were inverted and incubated in an incubator at 37 °C for 2 days. The results were observed, and finally, the average value of the results was taken. The determination of the microscopic structures referred to the method proposed by Stratakos et al. [11], with some modifications made according to the experimental design. Specifically, 2 mL of pumpkin juice under different ultrasonic powers was mixed with 2 mL of distilled water. Then, 20 μL of the mixture was transferred onto a glass slide and stained with an equal volume of 0.1% toluidine blue solution for 90 s. After that, the sample was observed under an inverted optical microscope Images were captured at a magnification of 10 times.

#### 2.2.12. The Aroma Substances in Pumpkin Juice Were Determined via Solid-Phase Microextraction Coupled with Gas Chromatography–Mass Spectrometry (GC-MS)

Solid-phase microextraction (SPME) coupled with gas chromatography–mass spectrometry was conducted according to the method described by Mulu Hagos et al. [11]. A total of 2 mL of the sample was accurately measured and transferred into a 20 mL volumetric flask. The vial was immediately sealed, placed in a 45 °C water bath, and pre-equilibration with gentle stirring was performed for 10 min, followed by extraction for 40 min. The sample was collected using an extraction needle. The extraction was carried out with an SPME fiber. Then, the extract was introduced into the injection port of the GC-MS and it was desorbed at 250 °C for 6 min. The chromatographic conditions were set as follows: J&W DB-5 quartz capillary column (length: 30 m, inner diameter: 0.25 mm, film thickness: 0.25); the initial column temperature was 45 °C and held for 3 min, then increased to 140 °C at a rate of 5 °C/min, and then increased to 220 °C at a rate of 10 °C/min and held for 5 min; the injection port temperature was 250 °C; the flow rate of the carrier gas (He) was 1.0 mL/min; and injection was adopted. The mass spectrometric conditions included an electron impact (EI) ion source, an electron energy of 70 eV, a transfer line temperature of 280 °C, an ion source temperature of 230 °C, and a mass scanning range of 33~350 u. The components in the pumpkin juice were qualitatively identified according to the retention index (RI) in the NIST 2017 library of the GC-MS system. For the quantitative analysis, the area normalization method was used [12,13,14], where the relative percentage to volatile compound was calculated by dividing the peak area of each component by the total peak area of all components. Each sample was tested three times repeatedly. It should be noted that the area normalization method assumes uniform response factors for all components, and its accuracy may be affected by differences in detector responses, which should be considered when interpreting the results.

#### 2.2.13. Determination of Sensory Evaluation

The evaluation was performed in the laboratory by 20 semi-trained panelists, including 10 females and 10 males aged 23 to 55 years. Before the sensory test, approximately 50 mL of pumpkin juice was poured into a colorless transparent 100 mL glass beaker, covered with a glass lid, labeled with unique three-digit random codes, and presented in random order. Panelists scored the pumpkin juice on a scale from extremely unappealing (1) to extremely appealing (10) for the following qualities: overall acceptability, color, aroma, taste, acidity, and turbidity. The final score was the average, and the specific scoring rules are shown in Table 3.

### 2.3. Data Processing

All the data obtained from this experiment were the results of three repetitions, and the results were expressed in the form of the mean ± standard deviation. The response surface optimization results were processed using Design-Expert 10.0 software. The charts were created using Origin 2021 and TBtools software (v2.309). The Waller–Duncan method in SPSS 22.0 software was employed for the significance analysis (*p* < 0.05).

## 3. Results

### 3.1. Results of the Response Surface Optimization of the Process for Yeast Fermentation of Pumpkin Juice

Through the preliminary single-factor experiments, the optimal ranges for each factor were determined as follows: the volume of pumpkin juice ranged from 15 to 25 milliliters, the inoculum size was between 2% and 4%, the fermentation time spanned from 0 to 2 days, and the fermentation temperature was within the range of 28–32 °C. Therefore, a response surface methodology experiment with four factors and three levels was designed to further optimize the fermentation process. The analysis was carried out using Design-Expert 10.0 software. According to the combined design scheme, the experimental design scheme is shown in Table 4 below, and the results of the analysis of variance are shown in Table 5 below.

### 3.2. Analysis Results of the Response Surface Methodology

As can be seen from Table 5, the regression model is significant (*p* < 0.0001), indicating that the model has a good fitting effect for this experiment. The coefficient of determination of this model, R_1_ = 0.9500, shows that there is a good correlation between the predicted values and the true values. The lack-of-fit term is small, and F > 0.05, which is not significant, indicating that the model has good simulation performance and can be used to analyze and predict the sensory evaluation results of pumpkin juice. According to the F values, the order of influence of various factors on the sensory quality of the product is C > A > B > D, that is, the fermentation time > the content of pumpkin juice > the inoculation amount of yeast > the temperature. A^2^ has an extremely significant influence on the sensory score of the product (*p* < 0.01). The optimal extraction conditions were determined by solving the obtained regression equation using Design Expert 10.0 software:

Sensory evaluation = 8.44 + 0.125 * A − 0.05 * B − 1.17 * C − 0.0417 * D + 0.00 * AB − 0.05 * AC − 0.375 * AD − 0.125 * BC + 0.075 * BD − 0.275 * CD − 1.0075 * A^2^ + 0.055 * B^2^ + 0.23 * C^2^ − 0.1075 * D^2^. The optimal fermentation process parameters were obtained as follows: the content of pumpkin juice was 18.11 mL, the fermentation temperature was 29.36 °C, the fermentation time was 1.48 days, and the inoculation amount was 3.54%. The predicted sensory score of the pumpkin juice was 7.70 points. Experiments were carried out according to the optimal process conditions to verify the accuracy of the model. A total of five parallel experiments were set up [15], and the average value of the sensory scores was 8.60 points. The actual value was higher than the predicted value, indicating that ultrasonic-assisted fermentation has great superiority and enormous potential, which proves that the experimental results have high accuracy and are basically consistent with the predicted values, indicating that the method is feasible. Considering the actual operability comprehensively, the fermentation process parameters were determined as follows: the content of pumpkin juice was 20 mL, the fermentation temperature was 30 °C, the fermentation time was 1 day, and the inoculation amount was 3%.

### 3.3. Analysis of the Interaction Effects of Various Factors on the Sensory Evaluation of Pumpkin Juice

**Figure 1:**
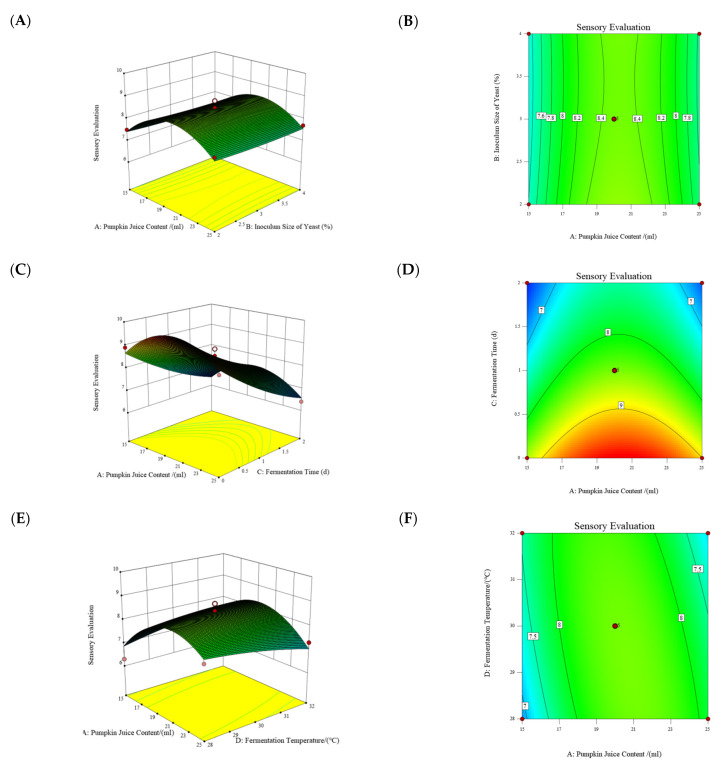
Response surface and contour plots illustrate the interactive effects of (**A**,**B**) the content of pumpkin juice and the inoculation size of yeast, (**C**,**D**) the content of pumpkin juice and the fermentation time, and (**E**,**F**) the content of pumpkin juice.

The regression equation was obtained using DesignExpert-8.0.6 software, and a series of response surface plots and contour plots were drawn, as shown in Figure 1, to conduct a study on the sensory evaluation of pumpkin juice, analyzing the influence of the interaction effects of various factors on the sensory evaluation. The slope of the response surface reflects the degree of influence of each factor on the sensory evaluation value. The steeper the slope, the greater the influence of the factor on the sensory evaluation. In the interaction plot between the content of pumpkin juice and the inoculation amount of yeast (Figure 1A), the change in the slope of the response surface can be observed to determine the strength of the influence of the two on the sensory evaluation. Similarly, in the interaction plots between the content of pumpkin juice and the fermentation time (Figure 1C), and between the content of pumpkin juice and the temperature (Figure 1E), the influence magnitude of the corresponding factors can be analyzed through the slope of the response surface. The shape of the contour lines reflects the degree of interaction between the factors. Elliptical contour lines indicate an obvious interaction effect, and the closer the shape is to a circle, the less significant the interaction effect. Observe the shapes of the contour lines in the interaction plots, such as between the content of pumpkin juice and the inoculation amount of yeast (Figure 1B), between the content of pumpkin juice and the temperature (Figure 1F), and between the content of pumpkin juice and the fermentation time (Figure 1D), to determine whether the interaction effect between the factors is obvious. For example, if the contour lines are elliptical, it indicates that the interaction effect of the corresponding two factors has a greater influence on the sensory evaluation; if they are close to a circle, the interaction effect has a smaller influence.

#### 3.3.1. Influence of the Interaction Between the Content of Pumpkin Juice and the Temperature on the Sensory Evaluation of Pumpkin Juice

Figure 1A shows the influence of the interaction between the content of pumpkin juice and the temperature on the sensory evaluation. When the fermentation temperature is constant, the sensory evaluation score first increases and then decreases with the increase in the content of pumpkin juice, indicating that there is a certain content of pumpkin juice at which the sensory evaluation score reaches the maximum. Similarly, as can be seen from Figure 1B, when the content of pumpkin juice is constant, with the increase in the fermentation temperature, the sensory evaluation score also first increases and then decreases, indicating that there is a certain fermentation temperature at which the sensory evaluation score is the highest. Therefore, under interactions between the fermentation temperature and the fermentation time, there must be an optimal sensory evaluation score.

#### 3.3.2. Influence of the Interaction Between the Content of Pumpkin Juice and the Inoculation Amount of Yeast on the Sensory Evaluation of Pumpkin Juice

Figure 1C shows the influence of the interaction between the content of pumpkin juice and the inoculation amount of yeast on the sensory evaluation. When the content of pumpkin juice is constant, the sensory evaluation score first increases and then decreases with the increase in the inoculation amount of yeast, indicating that there is a certain inoculation amount of yeast at which the sensory evaluation score is the highest. Similarly, as can be seen from Figure 1D, when the inoculation amount of yeast is constant, with the increase in the content of pumpkin juice, the sensory evaluation score also first increases and then decreases, indicating that there is a certain content of pumpkin juice at which the sensory evaluation score reaches the maximum. Therefore, under the interaction between the content of pumpkin juice and the inoculation amount of yeast, there must be an optimal combination that results in the highest sensory evaluation score.

#### 3.3.3. Influence of the Interaction Between the Content of Pumpkin Juice and the Fermentation Time on the Sensory Evaluation of Pumpkin Juice

Figure 1E shows the influence of the interaction between the content of pumpkin juice and the fermentation time on the sensory evaluation. When the content of pumpkin juice is constant, the sensory evaluation score first increases and then decreases with the increase in the fermentation time, indicating that there is a certain fermentation time at which the sensory evaluation score is the highest. Similarly, as can be seen from Figure 1F, when the fermentation time is constant, with the increase in the content of pumpkin juice, the sensory evaluation score also first increases and then decreases, indicating that there is a certain fermentation time at which the sensory evaluation score reaches the maximum. Therefore, under interactions between the content of pumpkin juice and the fermentation time, there must be an optimal sensory evaluation score.

### 3.4. Influence of Ultrasonic Treatment on the Total Acid Content of Pumpkin Juice

**Figure 2:**
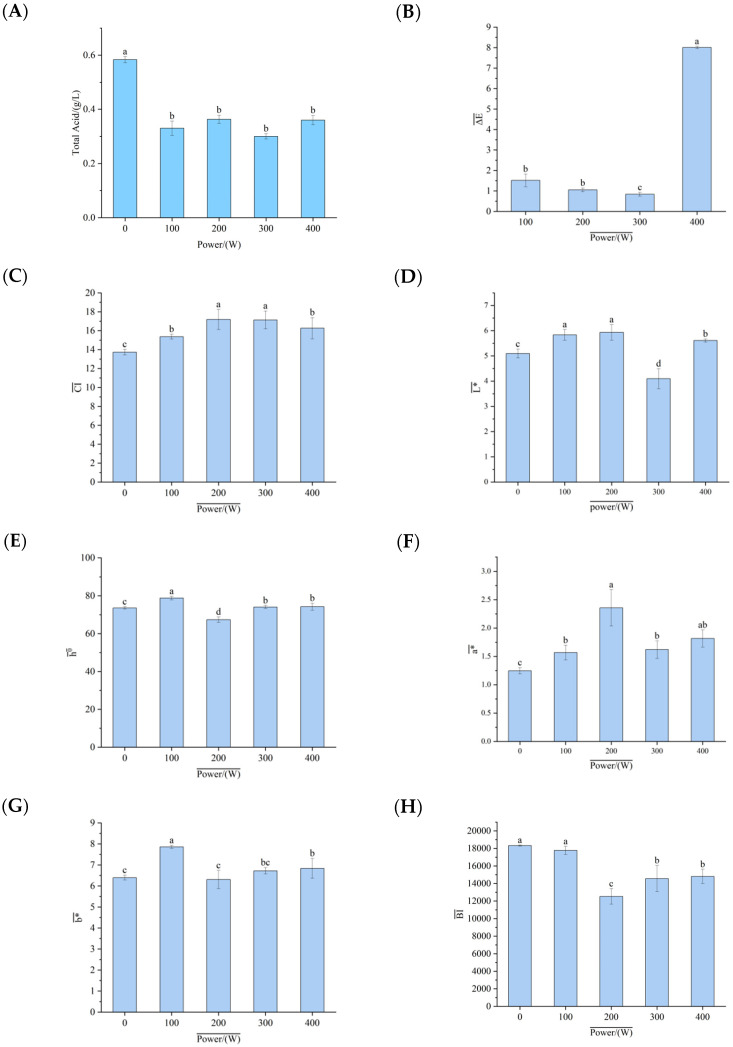

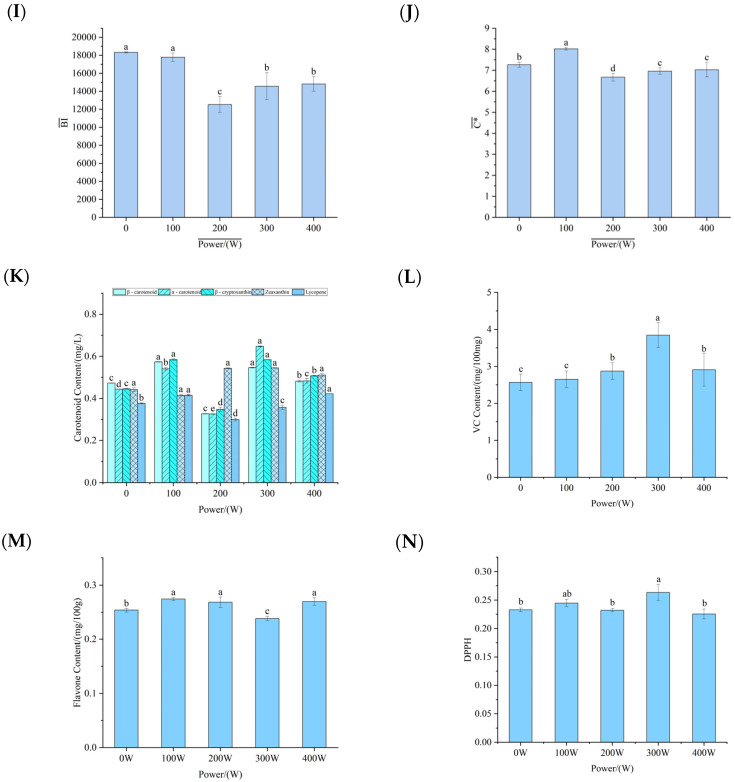
The influence of different ultrasonic powers on the physicochemical properties of fermented pumpkin juice. (**A**): The influence of ultrasonic treatment on the total acid content of pumpkin juice. (**B**–**J**): The influence of ultrasonic treatment on the color of pumpkin juice. (**K**): The influence of ultrasonic treatment on the carotenoids in pumpkin juice. (**L**): The influence of ultrasonic treatment on the ascorbic acid (VC) in pumpkin juice. (**M**): The influence of ultrasonic treatment on the flavonoid content in pumpkin juice. (**N**): The influence of ultrasonic treatment on the antioxidant activity of pumpkin juice.

The total acid content in pumpkin juice was a major factor affecting the sensory quality of pumpkin juice. When ultrasonic waves propagated in a liquid, phenomena such as cavitation, mechanical vibration, and thermal effects occurred. These physical effects might have changed the structure or distribution of acid molecules in the food, thereby influencing the total acid content [16]. As shown in Figure 2A, compared with the untreated group, the total acid content in the pumpkin juice decreased after treatment with ultrasonic waves of different powers. This result aligned with the findings of Kesavan et al. [17], who reported that ultrasonic treatment could reduce the total acid content in fruit juices. The relatively high total acid content in the non-ultrasonic-assisted fermented pumpkin juice might have negatively impacted the sensory properties of yeast-fermented pumpkin juice. Initially, ultrasonic treatment caused a significant decrease in the total acid content of pumpkin juice. However, as the ultrasonic power increased, the enhanced cavitation and mechanical effects led to complex changes [18]. On the one hand, the increased energy from higher-power ultrasonic waves might have disrupted certain chemical bonds or molecular interactions that kept acidic substances in a bound state within the food matrix, releasing some of the originally bound acidic substances and increasing the number of free acids. But on the other hand, other concurrent physical and chemical processes might have counteracted or modified this increase, resulting in the total acid content of the ultrasonic-treated pumpkin juice showing a slow upward trend after the initial decrease. Overall, this indicated that ultrasonic treatment could regulate the total acid content in pumpkin juice, and that such regulation was beneficial for improving the sensory properties of pumpkin juice after yeast fermentation and promoting the fermentation process of pumpkin juice by yeast.

### 3.5. Influence of Ultrasonic-Assisted Yeast Fermentation on the Color of Pumpkin Juice

Color is a key indicator that affects the consumer acceptability of food [19]. As can be seen from Figure 2B–J, for the pumpkin juice treated with ultrasonic waves, the L* value hardly changed. It only increased slightly after being treated at a power of 400 W, but the change was still not significant. When Brandão [20] et al. studied the color changes in blueberries, they found that regardless of the amplitude, ultrasonic treatment did not affect the L* value results of blueberry juice. The a* and b* values of the pumpkin juice after ultrasonic treatment also showed little difference from those of the control group, with little color change. The color difference the value ΔE between the two was less than 1.5, indicating that there was no significant color difference between the two samples. This result fully demonstrates that the pumpkin juice fermented by yeast after ultrasonic treatment can maintain its color extremely effectively, keeping it in a state similar to the color of natural pumpkin juice. Bhutkar et al. [6] found in their research that the total color difference between kiwifruit juice after thermo-ultrasonic treatment and before treatment was small, which is consistent with this conclusion. When the pumpkin juice is treated with ultrasonic waves, the water content inside the cells is reduced through the cavitation effect [21]; in this process, the dissolved oxygen content in the pumpkin juice decreases, and the reduction in the dissolved oxygen content plays an important inhibitory role in the enzymatic browning process of the pumpkin juice [22]. Enzymatic browning is one of the key factors leading to color changes in fruit and vegetable products. When the oxygen content decreases, it is difficult for the relevant enzymatic reactions to proceed fully, thus keeping the color of the pumpkin juice basically stable. This color stability is of great significance for the pumpkin juice fermented by yeast. It can ensure that the fermented pumpkin juice is more attractive in terms of color, and more in line with consumers’ expectations of the color of pumpkin juice. Furthermore, it can play a positive role in market promotion and product quality improvement. This is consistent with the research findings of Khandpur et al. [23], who discovered that the cavitation effect of ultrasonic waves leads to a decrease in the activity of polyphenol oxidase (PPO), thus better maintaining the color of orange juice.

### 3.6. Influence of Ultrasonic-Assisted Yeast Fermentation on the Carotenoids in Pumpkin Juice

The influence of ultrasonic-assisted yeast fermentation on the carotenoids in pumpkin juice is shown in Figure 2K. The results indicated that the content of carotenoids exhibited an obvious increasing trend as the ultrasonic power increased. However, when the ultrasonic power reached 400 W, the content of carotenoids decreased slightly. When treated at a power of 300 W, the measured carotenoid contents were 133.77%, 158.39%, 156.33%, 158.38%, and 86.45% in the control group (untreated group). This increase was mainly because ultrasonic treatment accelerated the rupture of the pumpkin cell wall. Breaking the barrier that previously restricted the release of carotenoids promoted the dissolution and release of carotenoids [24]. It is worth noting that, compared with the untreated group, the overall content of carotenoids still showed an upward trend. This finding aligned with the results reported by Fernandes et al. [25], who discovered that although carotenoids underwent chemical degradation, their content remained higher than in the untreated juice. Based on the experimental results, ultrasonic-treated pumpkin juice has certain advantages for subsequent processing. When the ultrasonic power was below 400 W, as the power increased, the dissolution rate of carotenoids rose. This might have been due to two main factors. First, the previous cell wall rupture had a cumulative effect. Second, the physical environment within the juice changed. Mechanical permeation enhanced the permeability of the solvent [26], and ultrasonic cavitation caused cell rupture, increasing the amount of substances inside the cells released into the solvent [27], which in turn raised the content of carotenoids.

### 3.7. Influence of Ultrasonic Treatment on the Ascorbic Acid (VC) in Pumpkin Juice

Ultrasonic waves had a certain influence on the VC content in pumpkin juice fermented by yeast. The cavitation effect and micro-jet generated by ultrasonic waves might have accelerated the interactions between ascorbic acid and the surrounding substances, such as contact with oxygen, enzymes, or other active ingredients, thus affecting its stability [28]. As can be seen from Figure 2L in the results, when treated with ultrasonic waves at a low power, there was no obvious change in the VC content in the pumpkin juice. When the ultrasonic power was 300 W, the VC content in the pumpkin juice fermented by yeast significantly increased by 1.381 mg/mL compared with that at 200 W. From the experiment, it is known that after ultrasonic treatment, it was beneficial to increase the VC content of the pumpkin juice after yeast fermentation. This is consistent with the research findings of Zhang et al. [29] that ultrasonic treatment increased the content of ascorbic acid. Kesavan et al. [17], when exploring the changes in the ascorbic acid content in fruit juices treated with ultrasonic waves and pasteurization, found that the ascorbic acid content in the fruit juice after ultrasonic treatment decreased from (37.21 ± 2.18) mg/100 mL to (27.10 ± 1.84) mg/100 mL, but that its retention rate was higher than that of the sample after pasteurization. The difference in the ascorbic acid content after ultrasonic treatment might have been related to the treatment conditions (temperature, time, ultrasonic power, etc.), and the specific mechanism of action still required further investigation.

### 3.8. Influence of Ultrasonic Treatment on the Flavonoid Content in Pumpkin Juice

The influence of ultrasonic treatment on the flavonoid content in pumpkin juice is shown in Figure 2M. The results indicate that ultrasonic waves have a certain impact on the flavonoid content in pumpkin juice fermented by yeast. Hao [30] optimized the conditions for the ultrasonic-assisted extraction of flavonoids from the leaves of Ixeris dentata var. mongolica and determined that the optimal ultrasonic extraction power for obtaining the highest total flavonoid content was 411.43 W, suggesting that the extraction of flavonoids is related to ultrasonic power. As the ultrasonic power increases, the flavonoid content in pumpkin juice shows a trend of increasing first and then decreasing. When the ultrasonic power is 100 W, the flavonoid content in the pumpkin juice reaches its peak, at 1.27 mg/mL (or another appropriate unit). This finding aligns with the research of RUIZ-DE ANDA D et al. [31], who found that ultrasonic treatment could increase the flavonoid content in juice samples via ultrasonic bath treatment. The mechanism behind this trend can be explained as follows: At lower ultrasonic power levels, the increasing ultrasonic power enhances the cavitation effect in the extraction system. The generated cavitation effect and oscillatory action correspondingly strengthen, increasing the degree of cell wall damage. This facilitates the dissolution of flavonoids inside the cells, thereby increasing the flavonoid yield. However, as the ultrasonic power continues to rise, both ultrasonic radiation and the thermal effect caused by ultrasonic treatment can induce changes in the structure of flavonoids. Excessively high ultrasonic power may damage the internal structure of flavonoids, leading to flavonoid loss and a subsequent decrease in the flavonoid yield. Notably, the flavonoid content in the pumpkin juice treated with 100 W ultrasonic waves is 112% of that in the untreated pumpkin juice, confirming the positive effect of moderate ultrasonic power on flavonoid extraction.

### 3.9. Influence of Ultrasonic-Assisted Yeast Fermentation on the Antioxidant Activity of Pumpkin Juice

The antioxidant effects of ultrasonic waves with different powers on the fermented pumpkin juice are shown in Figure 2N. In this experiment, the ability of pumpkin juice to scavenge free radicals was used as a measurement index to reflect the changing trend of its antioxidant activity after ultrasonic treatment. The experimental results clearly show that the antioxidant activity of pumpkin juice exhibits an upward trend with the increase in ultrasonic power, with only a slight decrease of 2.99% observed at 400 W. However, compared with the untreated pumpkin juice, this value does not show a significant difference. This phenomenon fully demonstrates that ultrasonic treatment can effectively retain the antioxidant activity of pumpkin juice to a large extent. This research result is consistent with the findings of Kobus et al. [32]. In their study, it was found that some enzymes, such as glucose oxidase, peroxidase (POD), and polyphenol oxidase (PPO), can be effectively inactivated via ultrasound. Meanwhile, the research conclusion obtained by Zhang et al. [29] that the antioxidant activity of pumpkin juice increases with the increase in power is also highly consistent with the results of this experiment. It is likely that the cavitation effect generated during the ultrasonic process plays a crucial role [33]. The cavitation effect can significantly enhance the extraction efficiency and utilization rate of phenolic compounds, thereby promoting improvements in the antioxidant properties of pumpkin juice. In addition, the factor of temperature also plays an important role in this process. An appropriate increase in temperature is beneficial in improving the solubility and diffusion rate of solutes, thus creating favorable conditions for the extraction of phenolic compounds. However, if the temperature is too high (exceeding 60 °C), it may cause the denaturation of phenolic compounds [11]; on the contrary, it will have a negative impact on its antioxidant performance. At the same time, some enzymes closely related to the oxidation process, such as PPO and POD involved in the enzymatic browning reaction, will be inactivated under the influence of ultrasonic cavitation, which also contributes to a certain extent to improvements in the antioxidant performance of pumpkin juice. In conclusion, through a comprehensive analysis of various factors, this study has deeply revealed the influence mechanism of ultrasonic waves on the antioxidant effect of fermented pumpkin juice, providing a valuable theoretical basis and data support for the optimization of pumpkin juice processing technology and research in the related fields.

### 3.10. Influence of Ultrasonic-Assisted Yeast Fermentation on the Microbial Flora and Microstructure of Pumpkin Juice

During the process of the ultrasonic treatment of pumpkin juice, the total number of colonies shows a significant regular change. That is, with the gradual increase in ultrasonic power, the total number of colonies in the pumpkin juice shows a continuous and obvious decreasing trend. This is consistent with the conclusion of Abid et al, [34], who found during the experiment that using ultrasonic (US) treatment alone on juice samples could significantly reduce the total number of colonies, as well as the number of yeasts and molds. After a detailed comparison of the sterilization effects at different power levels, it was found that when the ultrasonic power reached 400 W, the sterilization effect on pumpkin juice reached the optimal level, and the sterilization rate was as high as 32% at this time. By further comprehensively considering all the experimental results, a clear trend can be observed: the greater the ultrasonic power, the more significant the sterilization effect in pumpkin juice. The reduction in the number of microorganisms in pumpkin juice may be due to the disruption of microbial cell membranes during the ultrasonic treatment process [35]. During the propagation of ultrasonic waves, the phenomenon of cavity collapse occurs due to the unique cavitation effect [36]. This cavity collapse will instantly trigger the formation of a high-temperature and high-pressure environment in the local area [37]. The synergistic effect of these two extreme factors, namely high temperature and high pressure, directly targets the structure of the cell walls and cell membranes of microorganisms, causing their structures to disintegrate. Consequently, it undermines the integrity and physiological functions of microbial cells, ultimately achieving the goal of sterilization.

**Figure 3:**
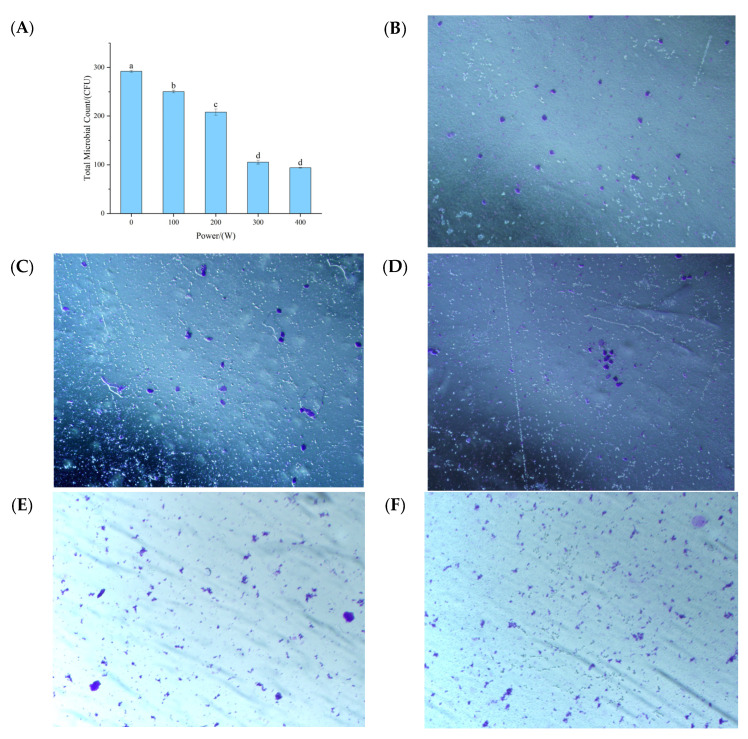
The influence of different ultrasonic power treatments on the total microbial count and microstructure of fermented pumpkin juice. (**A**): Effects of ultrasonic treatment on the number of microorganisms in pumpkin juice. (**B**): Microscopic structure of fermented pumpkin juice without ultrasonic treatment. (**C**): Microscopic structure of fermented pumpkin juice with ultrasonic treatment at a power of 100 W. (**D**): Microscopic structure of fermented pumpkin juice with ultrasonic treatment at a power of 200 W. (**E**): Microscopic structure of fermented pumpkin juice with ultrasonic treatment at a power of 300 W. (**F**): Microscopic structure of fermented pumpkin juice with ultrasonic treatment at a power of 400 W.

As can be seen from Figure 3A, the degree of agglomeration of the particles without ultrasonic treatment is slightly higher, which is also evident when the ultrasonic treatment is carried out at relatively low powers of 100 W and 200 W (Figure 3B,C). This indicates that lower power does not cause obvious changes in the particles in the pumpkin juice. However, when the ultrasonic power reaches 300 W and above, the particles are significantly crushed (Figure 3D,E), resulting in the rupture of the cells in the pumpkin juice due to cavitation. It is very likely that at a higher amplitude, the collapsing bubbles become more intense, and that the size of the bubbles is proportional to the amplitude of the ultrasonic waves [38]. This is consistent with the findings of Santos et al. [39], who discovered that low power does not cause significant changes in the morphology of the juice, but that the juice particles are crushed under high-power treatment. The study by Lepaus et al. [40] found that the orange–carrot blended juice treated with ultrasound showed an increase in the number of smaller particles, which is also in line with the results of this experiment. Wang et al. [41] found through their research that with the increase in ultrasonic time and power (>300 W) for mango juice, there were more particles with smaller particle sizes (decreasing from approximately 100 μm to 2.0 μm), which is also consistent with the results of this experiment.

### 3.11. Influence of Ultrasonic Treatment on the Aroma of Pumpkin Juice

**Figure 4:**
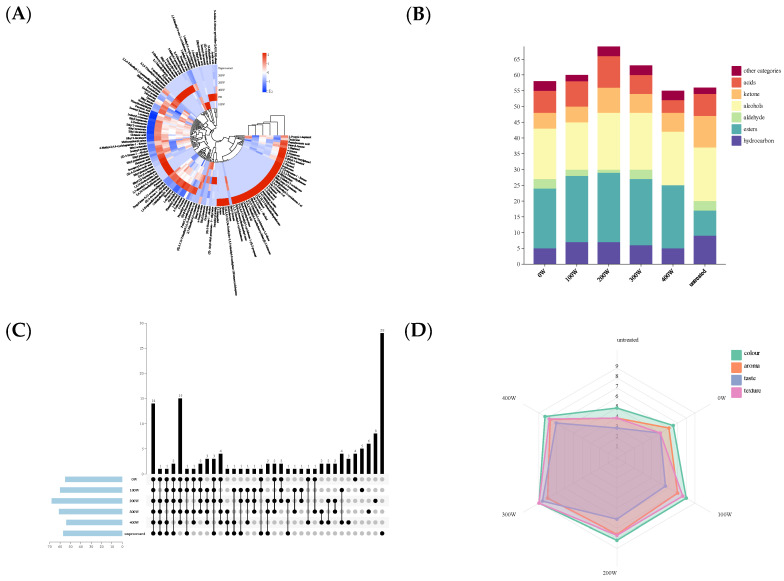
The influence of ultrasonic treatment on the types of aroma components in pumpkin juice (**A**–**C**). Sensory evaluation of pumpkin juice treated with ultrasonic waves of different powers (**D**). (**A**): Heat map of the influence of ultrasonic treatment with different powers on the types of aroma components in pumpkin juice. (**B**): Bar chart of the influence of ultrasonic treatment with different powers on the types of aroma components in pumpkin juice. (**C**): Venn diagram of the influence of ultrasonic treatment with different powers on the types of aroma components in pumpkin juice. (**D**): Radar chart of the sensory evaluation results of pumpkin juice treated with ultrasonic waves of different powers.

The results of the influence of ultrasonic waves on the aroma components of pumpkin juice are shown in Figure 4A–C. After ultrasonic treatment, 127 volatile flavor components were identified in the pumpkin juice fermented by yeast through gas chromatography. Among them, 14 volatile substances are the common flavors of different treatment groups, all of which are characteristic aroma profiles of pumpkin juice. This indicates that ultrasonic treatment (US) has little impact on the natural flavor of pumpkin juice. Ultrasonic treatment has newly generated a variety of substances such as acetic acid, eicosane, and isopropyl caprate in the aroma of the pumpkin juice fermented by yeast, indicating that ultrasonic treatment can cause the generation of new aroma components in the pumpkin juice fermented by yeast. This is consistent with the findings of Suo [1] et al. that ultrasonic treatment can lead to the formation of new compounds or the disappearance of compounds found in untreated samples. Sun et al. [42] compared the enzyme-treated orange juice with the fresh, untreated orange juice and found that the enzyme-treated one contained 15 new aroma compounds, while 3 aroma compounds disappeared.

In this experiment, the main volatile substances in the pumpkin juice fermented by yeast are esters, alcohols, and ketones. Hydrocarbons have a relatively high threshold and no special odor. Aldehydes usually have a fresh, fruity, and fatty aroma, with a low threshold, so they play a crucial role in flavor development. Aldehydes are mainly produced via the oxidative degradation of unsaturated fatty acids. After ultrasonic treatment, the overall content of aldehydes in the aroma of the pumpkin juice fermented by yeast shows a downward trend. The decrease in the content of aldehydes indicates that autoxidation and enzymatic oxidation may be involved [43]. Alcohols are mainly generated via the thermal oxidation of lipids. Alcohol compounds have low odor activity and exist in the form of glycoside conjugates in fruits [44]. After fermentation by yeast, the number of alcohol substances in the pumpkin juice increases, and the relative content increases by 344.64% compared with the untreated group. This indicates that ultrasonic treatment is conducive to the generation of new aroma components in the pumpkin juice fermented by yeast. After ultrasonic treatment, the content of esters in pumpkin juice increased significantly. This is consistent with the findings of Zou et al. [45], who discovered that the cavitation mixing effect generated by ultrasonic waves can increase the chances of successful collisions between alcohols and carboxylic acids and promote the formation of esters. In the early stage of ultrasonic treatment, the content of esters decreased. This may be because the thermal effect caused by ultrasonic waves increased the local temperature, leading to a reduction in the volatilization of esters. At the same time, the instantaneous high pressure generated by ultrasonic waves caused the hydrolysis of esters [13]. The content of ketone substances in the aroma of pumpkin juice after ultrasonic treatment increased slightly, indicating that ultrasonic waves have a promoting effect on the generation of ketone substances [46]. After ultrasonic treatment, the content of acidic substances in the aroma of pumpkin juice increased significantly. Guo et al. [47] optimized the optimal technological parameters for enriching polyphenols in black highland barley through single-factor experiments and the Box–Behnken response surface method for ultrasonic wave-assisted germination treatment. On this basis, they compared the differences in the phenolic acid composition of black highland barley before and after ultrasonic wave-assisted germination treatment. The results showed that the content of acidic substances in the samples treated via ultrasonic wave-assisted germination was significantly higher than that in the group without ultrasonic treatment.

### 3.12. Sensory Evaluation of Pumpkin Juice Fermented by Yeast with Ultrasonic Assistance

In this study, a sensory evaluation was conducted on pumpkin juice fermented by yeast with ultrasonic assistance. With the help of Figure 4D, the performance in four dimensions, namely color, aroma, taste, and texture, under different treatment conditions (represented by power levels from 0 W to 400 W and the untreated sample) was demonstrated. Color (Green): As can be seen from the radar chart, the color scores fluctuate under different power treatments. Compared with the untreated sample, the color scores of some power treatments improved, while those of others decreased, without showing a simple increasing or decreasing pattern. This indicates that the influence of ultrasonic power on the color of pumpkin juice is relatively complex. It may be because under the action of ultrasonic waves with different powers, the pigment substances inside the pumpkin juice have undergone physical or chemical changes to varying degrees, such as degradation, the isomerization of pigments, or their combination with other components. Aroma (Orange): The aroma scores vary significantly under different power conditions. As the power changes, the aroma scores rise and fall. This shows that ultrasonic power has a complex impact on the generation, volatilization, and interaction of the aroma components in pumpkin juice. It is likely that the cavitation effect and mechanical effect [48] of ultrasonic waves, etc., have promoted or inhibited the synthesis and release of aroma substances, altering the types and contents of aroma components. Taste (Blue): The taste scores show a trend of first increasing and then decreasing, or fluctuating with the change in power. This indicates that the influence of power on the taste of pumpkin juice is not unidirectional. It may be that the action of ultrasonic waves has affected the transformation and balance of flavor substances such as sugars and acids in the pumpkin juice. For example, the power may have promoted the hydrolysis of sugars or the generation of acids, thus changing the taste. Texture (Pink): The texture scores also vary to different extents under different power levels. The power may affect the texture of pumpkin juice by influencing its microstructure, such as particle size and viscosity [1]. It is likely that the mechanical action of ultrasonic waves causes the depolymerization or recombination of macromolecular substances in the pumpkin juice, changing the physical properties of the system. Ultrasonic waves with different powers, when assisting yeast fermentation, have a significant impact on the color, aroma, taste, and texture of pumpkin juice, and the impact is not a simple linear relationship. In actual production, to obtain pumpkin juice with the best sensory quality, it is necessary to precisely control the ultrasonic power and comprehensively consider the balance of various sensory dimensions [49]. Since the performance of different sensory dimensions varies under different power levels, it is difficult to determine a universal optimal power. It is necessary to further optimize the power conditions according to the specific emphasis on each sensory dimension required by the product, so as to improve the overall sensory acceptability of the pumpkin juice.

## 4. Conclusions

Through a series of single-factor analysis and response surface methodology optimization experiments, this study successfully determined the optimal fermentation process parameters for pumpkin juice: the content of pumpkin juice is 20 mL, the fermentation temperature is 30 °C, the fermentation time is 1 day, and the inoculation amount is 3%. Based on these parameters, the predicted sensory score of the pumpkin juice is as high as 8.5288 points. Compared with the fermented pumpkin juice without ultrasonic treatment, ultrasonic treatment shows significant advantages in improving the color, total acid content, and aroma components of pumpkin juice. In particular, when the ultrasonic power is set at 400 W, its sterilization effect on the microorganisms in the medium is the best, significantly reducing the number of microbial colonies in the pumpkin juice and meeting the microbial safety standards required by the commercial sector, thus ensuring the high quality and stability of the pumpkin juice. Ultrasonic waves with relatively high power (300 W) can effectively inactivate the activities of polyphenol oxidase and peroxidase, thereby reducing the enzymatic browning phenomenon of pumpkin juice. At the same time, they can retain the antioxidant active ingredients and vitamins in the pumpkin juice well. In terms of the natural biological activity in pumpkin juice, ultrasonic treatment not only effectively retains these active ingredients, but also reduces the particle size by disrupting the cell structure, improving the uniformity and taste of the pumpkin juice. This observation is consistent with the phenomena that the cell structure is damaged under the microscopic structure and the fragments of the cell wall decrease as the ultrasonic power increases. Microscopic analysis further reveals the mechanism by which ultrasonic treatment increases the carotenoid content by disrupting the cell structure. These results indicate that ultrasonic treatment has a positive impact on the fermentation process of pumpkin juice, especially in optimizing the fermentation process parameters and maintaining the product quality. Therefore, using ultrasonic treatment to assist the fermentation of fruit and vegetable juices by yeast not only meets the microbial standards and taste requirements of edible products, but also maximally retains the nutritional value of fruit and vegetable juices. At the same time, we believe that ultrasonic-assisted fermentation can protect the environment by shortening the fermentation cycle, improving raw material utilization, reducing energy and resource consumption, decreasing chemical reagent usage, and minimizing pollutant emissions, providing new ideas for improving the efficiency of the food industry.

## Figures and Tables

**Table 1:** Response surface method factors and levels.

Horizontal Value	A: Pumpkin Juice Content/mL	B: Volume/%	C: Fermentation Time/d	D: Fermentation Temperature/°C
−1	15	2	0	28
0	20	3	1	30
1	25	4	2	32

**Table 2:** Sensory evaluation of ultrasonic-assisted yeast fermentation of pumpkin juice under different fermentation conditions.

Project	Sample	Score/(Points)
Pumpkin Juice Content	10 mL	7.3 ± 0.6
	15 mL	7.1 ± 0.6
	20 mL	9.8 ± 0.8
	25 mL	6.9 ± 0.6
	30 mL	7.5 ± 0.7
Yeast Inoculation Amount	1%	6.8 ± 0.6
	2%	7.3 ± 0.6
	3%	8.8 ± 0.8
	4%	7.5 ± 0.6
	5%	6.3 ± 0.6
Fermentation Time	0 d	8.0 ± 0.7
	1 d	8.8 ± 0.8
	2 d	7.8 ± 0.7
	3 d	7.3 ± 0.7
	4 d	6.1 ± 0.6
Fermentation Temperature	26 °C	7.0 ± 0.6
	28 °C	8.1 ± 0.8
	30 °C	8.3 ± 0.8
	32 °C	7.3 ± 0.7
	34 °C	6.8± 0.6

**Table 3:** Score rules for sensory evaluation.

Project	Scoring Scale	Score
**Color (10 points)**	The color is even and the pumpkin juice is golden yellow.	8–10 points
	The color is even and the pumpkin juice is yellow.	4–7 points
	The color is uneven and variegated and the pumpkin juice is light yellow.	0–3 points
**Aroma (10 points)**	Pumpkin flavor and fermented flavor, no spoilage.	8–10 points
	A little pumpkin flavor and fermented flavor, no spoilage.	4–7 points
	No pumpkin flavor or fermented flavor, with other pungent flavors.	0–3 points
**Taste (10 points)**	Mellow and delicate taste, moderately sweet and sour.	8–10 points
	The taste is mellow, the texture is slightly rough, slightly sour or slightly sweet.	4–7 points
	Poor taste, rough texture, sour or sweet.	0–3 points
**Organizational status (10 points)**	Uniform organization, no stratification, no clots, and flocculent.	8–10 points
	Uniform tissue with slight stratification, clots, and flocculent.	4–7 points
	Uneven tissue, obviously layered, with clots, and flocculent.	0–3 points

**Table 4:** Response surface experimental design scheme.

Experiment	A: Pumpkin Juice Content/mL	B: Volume/%	C: Fermentation Time/d	Fermentation Temperature/°C	Sensory Evaluation Score
1	20	3	0	32	7.1
2	25	3	2	30	7.0
3	25	3	1	28	6.3
4	20	4	1	28	8.5
5	15	3	1	28	8.2
6	25	4	1	30	9.8
7	15	3	2	30	8.5
8	25	2	1	30	7.4
9	20	4	1	32	8.5
10	25	3	0	30	7.6
11	20	3	1	30	6.7
12	20	3	1	30	9.8
13	20	3	1	30	8.8
14	20	3	1	30	8.3
15	20	4	2	30	7.5
16	15	4	1	30	8.4
17	20	2	1	28	9.8
18	20	3	2	32	8.4
19	20	2	0	30	7.4
20	15	3	1	32	8.9
21	15	3	0	30	8.8
22	25	3	1	32	8.1
23	20	3	2	28	9.8
24	20	3	0	28	7.3
25	20	2	1	32	6.4
26	20	2	2	30	7.7
27	20	4	0	30	7.8
28	20	3	1	30	7.7
29	15	2	1	30	8.3

**Table 5:** Response surface test results.

Source of Variance	Sum of Squares	Degrees of Freedom	Mean	F Value	*p* Value
Model	25.53	14	1.82	19.01	<0.0001
A	0.19	1	019	1.95	0.1838
B	0.03	1	0.03	0.31	0.5848
C	16.33	1	16.33	170.29	<0.0001
D	0.021	1	0.021	0.22	0.6483
AB	3.553 × 10^−15^	1	3.553 × 10^−15^	3.704 × 10^−14^	1
AC	0.01	1	0.01	0.1	0.7516
AD	0.56	1	0.56	5.86	0.0296
BC	0.062	1	0.062	0.65	0.433
BD	0.022	1	0.022	0.23	0.6356
CD	0.3	1	0.3	3.15	0.0975
A^2^	6.58	1	6.58	68.64	<0.0001
B^2^	0.02	1	0.02	0.2	0.658
C^2^	0.34	1	0.34	3.58	0.0794
D^2^	0.075	1	0.075	0.78	0.3916
Residual	1.34	14	0.096		
Lack of Fit	1.13	10	0.11	2.13	0.2421
Pure Error	0.21	4	0.053		
Cor Total	26.87	28			
R^2^	0.9500				
R^2^_Adj_	0.9000				

## Data Availability

The original contributions presented in the study are included in the article, further inquiries can be directed to the corresponding author.

## References

[1] Suo G., Zhou C., Su W., Hu X. (2022). Effect of Ultrasonic Non-Thermal Sterilization on the Volatile Components of Pumpkin Juice. J. Comp. Methods Sci. Eng..

[2] Huezo L., Shah A., Michel Jr. F.C. (2019). Effects of Ultrasound on Fermentation of Glucose to Ethanol by Saccharomyces Cerevisiae. Fermentation.

[3] Salehi F. (2020). Physico-Chemical Properties of Fruit and Vegetable Juices as Affected by Pulsed Electric Field: A Review. Int. J. Food Prop..

[4] Keșa A.-L., Pop C.R., Mudura E., Salanță L.C., Pasqualone A., Dărab C., Burja-Udrea C., Zhao H., Coldea T.E. (2021). Strategies to Improve the Potential Functionality of Fruit-Based Fermented Beverages. Plants.

[5] Rawson A., Patras A., Tiwari B.K., Noci F., Koutchma T., Brunton N. (2011). Effect of Thermal and Non Thermal Processing Technologies on the Bioactive Content of Exotic Fruits and Their Products: Review of Recent Advances. Food Res. Int..

[6] Bhutkar S., Brandão T.R.S., Silva C.L.M., Miller F.A. (2024). Application of Ultrasound Treatments in the Processing and Production of High-Quality and Safe-to-Drink Kiwi Juice. Foods.

[7] Provesi J.G., Dias C.O., Amante E.R. (2011). Changes in Carotenoids during Processing and Storage of Pumpkin Puree. Food Chem..

[8] Barba F.J., Esteve M.J., Frigola A. (2010). Ascorbic Acid Is the Only Bioactive That Is Better Preserved by High Hydrostatic Pressure than by Thermal Treatment of a Vegetable Beverage. J. Agric. Food Chem..

[9] Kwaw E., Ma Y., Tchabo W., Apaliya M.T., Sackey A.S., Wu M., Xiao L. (2018). Impact of Ultrasonication and Pulsed Light Treatments on Phenolics Concentration and Antioxidant Activities of Lactic-Acid-Fermented Mulberry Juice. LWT.

[10] Xu H., Feng L., Deng Y., Chen L., Li Y., Lin L., Liang M., Jia X., Wang F., Zhang X. (2023). Change of Phytochemicals and Bioactive Substances in Lactobacillus Fermented Citrus Juice during the Fermentation Process. LWT.

[11] Stratakos A.C., Delgado-Pando G., Linton M., Patterson M.F., Koidis A. (2016). Industrial Scale Microwave Processing of Tomato Juice Using a Novel Continuous Microwave System. Food Chem..

[12] Hagos M., Yaya E.E., Chandravanshi B.S., Redi-Abshiro M. (2022). Analysis of Volatile Compounds in Flesh, Peel and Seed Parts of Pumpkin (Cucurbita Maxima) Cultivated in Ethiopia Using Gas Chromatography-Mass Spectrometry (GC-MS). Int. J. Food Prop..

[13] Zou Y., Kang D., Liu R., Qi J., Zhou G., Zhang W. (2018). Effects of Ultrasonic Assisted Cooking on the Chemical Profiles of Taste and Flavor of Spiced Beef. Ultrason. Sonochem..

[14] Kong Y., Sun L., Wu Z., Li Y., Kang Z., Xie F., Yu D. (2023). Effects of Ultrasonic Treatment on the Structural, Functional Properties and Beany Flavor of Soy Protein Isolate: Comparison with Traditional Thermal Treatment. Ultrason. Sonochem..

[15] Rheem S. (2023). Optimizing Food Processing through a New Approach to Response Surface Methodology. Food Sci Anim Resour.

[16] Zhang L., Lin Z., Zeng L., Zhang F., Sun L., Sun S., Wang P., Xu M., Zhang J., Liang X. (2022). Ultrasound-Induced Biophysical Effects in Controlled Drug Delivery. Sci. China Life Sci..

[17] Kesavan R.K., Gogoi S., Nayak P.K. (2023). Influence of Thermosonication and Pasteurization on the Quality Attributes of Kutkura (Meyna Spinosa) Juice. Appl. Food Res..

[18] Custodio-Mendoza J.A., Pokorski P., Aktaş H., Kurek M.A. (2024). Rapid and Efficient High-Performance Liquid Chromatography-Ultraviolet Determination of Total Amino Acids in Protein Isolates by Ultrasound-Assisted Acid Hydrolysis. Ultrason. Sonochemistry.

[19] Ayustaningwarno F., Fogliano V., Verkerk R., Dekker M. (2021). Surface Color Distribution Analysis by Computer Vision Compared to Sensory Testing: Vacuum Fried Fruits as a Case Study. Food Res. Int..

[20] Brandão T.R.S., Silva C.L.M., Miller F.A. (2024). The Power of Thermosonication on Quality Preservation and Listeria Control of Blueberry Juice. Foods.

[21] Kutlu N., Pandiselvam R., Kamiloglu A., Saka I., Sruthi N.U., Kothakota A., Socol C.T., Maerescu C.M. (2022). Impact of Ultrasonication Applications on Color Profile of Foods. Ultrason. Sonochem..

[22] Shi J., Wang S., Yao J., Cui M., Hu B., Wang J., Li F., Wang S., Tong R., Li M. (2024). Ultrasound Treatment Alleviates External Pericarp Browning and Improves Fruit Quality of Pomegranate during Storage. J. Sci. Food Agric..

[23] Khandpur P., Gogate P.R. (2015). Understanding the Effect of Novel Approaches Based on Ultrasound on Sensory Profile of Orange Juice. Ultrason. Sonochem..

[24] Buniowska M., Arrigoni E., Znamirowska A., Blesa J., Frígola A., Esteve M.J. (2019). Liberation and Micellarization of Carotenoids from Different Smoothies after Thermal and Ultrasound Treatments. Foods.

[25] Fernandes F.A.N., Santos V.O., Gomes W.F., Rodrigues S. (2023). Application of High-Intensity Ultrasound on Acerola (Malpighia Emarginata) Juice Supplemented with Fructooligosaccharides and Its Effects on Vitamins, Phenolics, Carotenoids, and Antioxidant Capacity. Processes.

[26] Ordóñez-Santos L.E., Velasco-Arango V.A., Hleap-Zapata J.I. (2022). Ultrasound-Assisted Extraction of Total Carotenoids in Papaya Epicarp and Its Application in Frankfurt Sausage. Ciênc. Agrotec..

[27] Sebdani M.M., Abbasi H. (2023). Green Extraction of Carotenoids from Pumpkin with Ultrasound-Assisted Method; Optimization Using Response Surface Methodology. Microchem. J..

[28] Zi-xuan G.U., Zheng-yu J.I.N., Bing-hua S.U.N., Yao-qi T. (2018). Preparation of Hyper-Branched Starch-Ascorbic Acid Inclusion Complex and Its Photo-Thermostability. Food Ferment. Ind..

[29] Zhang M., Zhou C., Ma L., Su W., Jiang J., Hu X. (2024). Influence of Ultrasound on the Microbiological, Physicochemical Properties, and Sensory Quality of Different Varieties of Pumpkin Juice. Heliyon.

[30] Hao J., Wang Z., Jia Y., Sun L., Fu Z., Zhao M., Li Y., Yuan N., Cong B., Zhao L. (2023). Optimization of Ultrasonic-Assisted Extraction of Flavonoids from Lactuca Indica L. Cv. Mengzao and Their Antioxidant Properties. Front. Nutr..

[31] Ruiz-De Anda D., Ventura-Lara M.G., Rodríguez-Hernández G., Ozuna C. (2019). The Impact of Power Ultrasound Application on Physicochemical, Antioxidant, and Microbiological Properties of Fresh Orange and Celery Juice Blend. Food Meas..

[32] Kobus Z., Osmólska E., Starek-Wójcicka A., Krzywicka M. (2023). Effect of High-Powered Ultrasound on Bioactive Compounds and Microbiological Stability of Juices—Review. Appl. Sci..

[33] Jiang Q., Zhang M., Xu B. (2020). Application of Ultrasonic Technology in Postharvested Fruits and Vegetables Storage: A Review. Ultrason Sonochem.

[34] Abid M., Jabbar S., Hu B., Hashim M.M., Wu T., Wu Z., Khan M.A., Zeng X. (2014). Synergistic Impact of Sonication and High Hydrostatic Pressure on Microbial and Enzymatic Inactivation of Apple Juice. LWT-Food Sci. Technol..

[35] Nadulski R., Kobus Z., Wilczyński K., Sobczak P., Panasiewicz M., Żukiewicz-Sobczak W., Szparaga A. (2019). Effect of Extraction Method and Thermosonication on Apple Juice Quality. Appl. Sci..

[36] Muñoz R., Viveros N., Bevilacqua A., Pérez M.S., Arévalo-Villena M. (2021). Effects of Ultrasound Treatments on Wine Microorganisms. Ultrason. Sonochem..

[37] Xu B., Azam S.M.R., Feng M., Wu B., Yan W., Zhou C., Ma H. (2021). Application of Multi-Frequency Power Ultrasound in Selected Food Processing Using Large-Scale Reactors: A Review. Ultrason. Sonochem..

[38] Foujdar R., Bera M.B., Chopra H.K. (2020). Optimization of Process Variables of Probe Ultrasonic-Assisted Extraction of Phenolic Compounds from the Peel of Punica Granatum Var. Bhagwa and It’s Chemical and Bioactivity Characterization. J. Food Process. Preserv..

[39] Santos J.C.C., Correa J.L.G., Furtado M.L.B., de Morais L.C., Borges S.V., de Oliveira C.R., de Resende J.V., de Oliveira L.F. (2024). Influence of Intensity Ultrasound on Rheological Properties and Bioactive Compounds of Araticum (*Annona Crassiflora*) Juice. Ultrason. Sonochem..

[40] Lepaus B.M., Santos A.K.P.d.O., Spaviero A.F., Daud P.S., de São José J.F.B. (2022). Stability Parameters during Refrigerated Storage and Changes on the Microstructure of Orange-Carrot Blend Juice Processed by High-Power Ultrasound. Front. Sustain. Food Syst..

[41] Wang J., Liu Q., Xie B., Sun Z. (2020). Effect of Ultrasound Combined with Ultraviolet Treatment on Microbial Inactivation and Quality Properties of Mango Juice. Ultrason. Sonochem..

[42] Sun Y., Xu Q., Peng W., Xue Y., Sun P. (2021). Synergistic Effects of Ultrasound and β-d-Glucosidase in Aroma of Orange Juice. J Food Sci..

[43] Xu B., Feng M., Chitrakar B., Cheng J., Wei B., Wang B., Zhou C., Ma H. (2023). Multi-Frequency Power Thermosonication Treatments of Clear Strawberry Juice: Impact on Color, Bioactive Compounds, Flavor Volatiles, Microbial and Polyphenol Oxidase Inactivation. Innov. Food Sci. Emerg. Technol..

[44] Biswas P., Paul S., Guin J. (2016). Aerobic Radical-Cascade Alkylation/Cyclization of α,β-Unsaturated Amides: An Efficient Approach to Quaternary Oxindoles. Angew. Chem. Int. Ed..

[45] Nikfardjam M.P., Maier D. (2011). Development of a Headspace Trap HRGC/MS Method for the Assessment of the Relevance of Certain Aroma Compounds on the Sensorial Characteristics of Commercial Apple Juice. Food Chem..

[46] Ji S.-J., Shen Z.-L., Gu D.-G., Wang S.-Y. (2004). An Efficient Synthesis of Ferrocenyl Substituted 1,5-Diketone and Cyclic α,β-Unsaturated Ketones under Ultrasound Irradiation. J. Organomet. Chem..

[47] Junling G.U.O., Jie Z., Wengang Z., Bin D., Xijuan Y. (2023). Process Optimization of Ultrasonic Co-germination for Enriching Black Highland Barley Polyphenols and Analysis of Phenolic Acid Composition. Sci. Technol. Food Ind..

[48] Ojha K.S., Mason T.J., O’Donnell C.P., Kerry J.P., Tiwari B.K. (2017). Ultrasound Technology for Food Fermentation Applications. Ultrason. Sonochem..

[49] Türkol M., Yıkmış S., Ganimet Ş., Gezer G.E., Abdi G., Hussain S., Aadil R.M. (2024). Optimization of Sensory Properties of Ultrasound-Treated Strawberry Vinegar. Ultrason. Sonochem..

